# Use of a national continuing medical education meeting to provide simulation-based training in temporary hemodialysis catheter insertion skills: a pre-test post-test study

**DOI:** 10.1186/s40697-014-0025-6

**Published:** 2014-10-14

**Authors:** Edward G Clark, James J Paparello, Diane B Wayne, Cedric Edwards, Stephanie Hoar, Rory McQuillan, Michael E Schachter, Jeffrey H Barsuk

**Affiliations:** Department of Medicine, Division of Nephrology, The Ottawa Hospital, 1967 Riverside Drive, Ottawa, Ontario K1H 7 W9 Canada; Department of Medicine, Division of Nephrology, Northwestern University Feinberg School of Medicine, 251 E Huron Street, Feinberg 16-738, Chicago, IL 60611 USA; Department of Medicine and Department of Medical Education, Northwestern University Feinberg School of Medicine, 251 E Huron Street, Feinberg 16-738, Chicago, IL 60611 USA; Department of Medicine, Division of Nephrology, The Toronto General Hospital, 8 N-840, 200 Elizabeth Street, Toronto, Ontario M5G 2C4 Canada; Department of Medicine, Division of Nephrology, Royal Jubilee Hospital, 1952 Bay Street, Victoria, BC V8R 1 J8 Canada; Department of Medicine, Northwestern University Feinberg School of Medicine, 251 E Huron Street, Feinberg 16-738, Chicago, IL 60611 USA

**Keywords:** Central venous catheterization, Mastery learning, Vascular access, Simulation-based education, Medical education, Temporary hemodialysis catheter, Non-tunneled hemodialysis catheter, Ultrasound, Clinical competence

## Abstract

**Background:**

Simulation-based-mastery-learning (SBML) is an effective method to train nephrology fellows to competently insert temporary, non-tunneled hemodialysis catheters (NTHCs). Previous studies of SBML for NTHC-insertion have been conducted at a local level.

**Objectives:**

Determine if SBML for NTHC-insertion can be effective when provided at a national continuing medical education (CME) meeting. Describe the correlation of demographic factors, prior experience with NTHC-insertion and procedural self-confidence with simulated performance of the procedure.

**Design:**

Pre-test – post-test study.

**Setting:**

2014 Canadian Society of Nephrology annual meeting.

**Participants:**

Nephrology fellows, internal medicine residents and medical students.

**Measurements:**

Participants were surveyed regarding demographics, prior NTHC-insertion experience, procedural self-confidence and attitudes regarding the training they received. NTHC-insertion skills were assessed using a 28-item checklist.

**Methods:**

Participants underwent a pre-test of their NTHC-insertion skills at the internal jugular site using a realistic patient simulator and ultrasound machine. Participants then had a training session that included a didactic presentation and 2 hours of deliberate practice using the simulator. On the following day, trainees completed a post-test of their NTHC-insertion skills. All participants were required to meet or exceed a minimum passing score (MPS) previously set at 79%. Trainees who did not reach the MPS were required to perform more deliberate practice until the MPS was achieved.

**Results:**

Twenty-two individuals participated in SBML training. None met or exceeded the MPS at baseline with a median checklist score of 20 (IQR, 7.25 to 21). Seventeen of 22 participants (77%) completed post-testing and improved their scores to a median of 27 (IQR, 26 to 28; p < 0.001). All met or exceeded the MPS on their first attempt. There were no significant correlations between demographics, prior experience or procedural self-confidence with pre-test performance.

**Limitations:**

Small sample-size and self-selection of participants. Costs could limit the long-term feasibility of providing this type of training at a CME conference.

**Conclusions:**

Despite most participants reporting having previously inserted NTHCs in clinical practice, none met the MPS at baseline; this suggests their prior training may have been inadequate.

## ABRÉGÉ

**Contexte:**

L’apprentissage assuré par la simulation est une méthode efficace pour former les résidents en néphrologie à insérer un cathéter d’hémodialyse non tunnellisé. Des études précédentes sur l’apprentissage assuré par la simulation pour l’insertion de cathéters non tunnellisés ont été effectuées à l’échelon local.

**Objectifs:**

Déterminer si l’apprentissage assuré par la simulation pour l’insertion de cathéters non tunnellisés peut être efficace lorsque les possibilités sont offertes lors d’une conférence nationale de formation médicale continue (FMC). Décrire la corrélation entre les facteurs démographiques, les expériences antérieures d’insertion de cathéters non tunnellisés, de même que l’assurance personnelle en matière de simulation de la procédure. Évaluer la perception des apprenants face à l’apprentissage assuré par la simulation dans le cadre d’une conférence nationale de FMC.

**Type d’étude:**

Prétest/post-test.

**Contexte:**

Réunion annuelle 2014 de la Société canadienne de néphrologie.

**Participants:**

Les résidents en néphrologie et en médecine interne et les étudiants en médecine.

**Mesures:**

On a effectué un sondage auprès des participants au sujet des caractéristiques démographiques, de leurs expériences antérieures d’insertion de cathéters non tunnellisés, de leur assurance personnelle et leur attitude par rapport à la formation reçue. Les compétences en matière d’insertion de cathéters non tunnellisés ont été évaluées selon une liste de contrôle en 28 points.

**Méthodes:**

Les participants ont subi un prétest de leurs compétences en matière d’insertion de cathéters non tunnellisés dans la veine jugulaire interne, à l’aide d’un simulateur de patient et d’une machine à échographie. Les participants ont ensuite suivi une séance de formation qui comprenait une présentation didactique et deux heures d’exercices sur le simulateur. Le jour suivant, ils ont subi un post-test de leurs compétences. Tous les participants devaient atteindre ou dépasser la note minimale de passage précédemment fixée à 79%. Ceux qui n’ont pas atteint cette note ont dû effectuer des exercices supplémentaires jusqu’à l’atteindre.

**Résultats:**

Vingt-deux personnes ont participé à la formation sur l’insertion de cathéters d’hémodialyse non tunnellisés. Aucun d’entre eux n’a atteint ou dépassé la note minimale de passage en premier lieu, pour une médiane de 20 (ÉI = écart interquartile, entre 7,25 et 21). Dix-sept des 22 participants (77%) ont terminé le post-test en améliorant leur note, pour une médiane de 27 (ÉI, entre 26 et 28; p < 0,0001). Tous ont atteint ou excédé la note de passage lors de leur premier essai. Il n’existe aucune corrélation significative entre les facteurs démographiques, l’expérience antérieure et l’assurance personnelle, d’une part, et les résultats du prétest, d’autre part. Les participants ont confirmé l’apport de la formation, et qu’elle devrait être intégrée à la formation postdoctorale en néphrologie.

**Limites de l’étude:**

Échantillonnage restreint et autosélection des participants. Le rapport coût-efficacité n’a pas été évalué. Les coûts pourraient limiter la faisabilité à long terme de la prestation de ce type de formation au cours de conférences de FMC.

**Conclusions:**

Bien que plusieurs participants aient rapporté posséder de l’expérience antérieure dans l’insertion de cathéters non tunnellisés en pratique clinique, aucun d’entre eux n’a atteint la note minimale de passage en premier lieu; ceci suggère que leur formation antérieure ait été inadéquate. Il est possible d’offrir des possibilités d’apprentissage assuré par la simulation pour l’insertion de cathéters d’hémodialyse non tunnellisés qui soit efficace dans le contexte d’une conférence nationale de formation médicale continue.

## What was known before

Simulation-based-mastery-learning (SBML) is an effective method to train nephrology fellows to insert temporary, non-tunneled hemodialysis catheters (NTHCs). Prior studies of SBML for NTHC-insertion have been conducted at a local level.

## What this adds

SBML for NTHC-insertion can be effective when provided in the setting of a continuing medical education (CME) conference.

## Background

The ability to competently insert a temporary non-tunneled hemodialysis catheter (NTHC) is a requirement of nephrology training in Canada [1]. Nonetheless, evidence suggests that many Canadian nephrology trainees do not feel adequately trained to perform the procedure [2]. This is important because improper NTHC-insertion is associated with mechanical and infectious complications that are serious and potentially avoidable [3-5].

Simulation-based-mastery-learning (SBML) is a rigorous form of competence-based education in which learners complete pre-testing, deliberate skills practice with feedback and post-testing until they meet or exceed a predetermined minimum passing score (MPS) [6,7]. Learners who do not initially achieve the MPS undergo further deliberate practice and are retested until they achieve the MPS. In mastery learning, practice time may vary between learners but educational outcomes are uniform. Use of this model (and a stringent MPS) assures that all learners demonstrate their competency in a simulated environment before performing the procedure during actual clinical care [6,7]. SBML has been shown to be effective for multiple clinical skills, including NTHC-insertion [8,9]; however, most previous studies of SBML procedural-training have occurred at the level of a single training program and no information is available regarding the use of SBML in a continuing medical education (CME) setting.

The Canadian Society of Nephrology (CSN) annual meeting provides a unique opportunity to gather current and future nephrology fellows for specialized training during its pre-conference educational program. As such, we sought to determine if SBML for internal jugular (IJ) NTHC-insertion would be effective in this CME setting. The specific aims of this study were to: 1) evaluate the feasibility and effectiveness of NTHC-insertion SBML sessions at the CSN annual meeting; 2) describe the correlations of demographic factors, prior experience with NTHC-insertion and procedural self-confidence with simulated performance of the procedure; and 3) assess learners’ perceptions of SBML as part of a national CME conference.

## Methods

### Study design

The study was a pre-test – post-test (before-after) design [10], assessing an NTHC-insertion SBML pre-conference course during the 2014 CSN annual meeting in Vancouver. Participants were Canadian medical trainees who registered for the CSN annual meeting pre-conference educational program or precourse. The precourse took place on April 24 and 25, 2014 in a conference room of the same hotel that hosted the CSN annual meeting. The Northwestern University Institutional Review Board (Chicago, IL, USA) and the Ottawa Hospital Research Ethics Board (Ottawa, ON, Canada) approved the study and all participants provided informed consent.

### Procedure

Study participants provided demographic information including age, gender, year of post-graduate medical training; prior NTHC-insertion clinical experience (number of prior IJ NTHC-insertions) and; a rating of their self-confidence to perform NTHC-insertion competently using a scale of 0-100 (0 = not confident, 100 = extremely confident). Subsequently, all participants underwent an NTHC-insertion clinical skills baseline examination (pre-test). Pre-test clinical skills examinations were conducted using a previously described IJ NTHC-insertion checklist [9]. Over approximately 2 hours, all participants together completed a lecture, video presentation and ultrasound training. Subsequently, groups of 10 or less sequentially underwent approximately 2 hours of deliberate NTHC skills practice using the simulator with directed feedback. Practice sessions involved two participants and one faculty member (JHB, EC, CE, RM, JJP, MS) with expertise in teaching and performing NTHC-insertion at each of the 5 practice-and-testing stations. Participants were scheduled to return the following day for a repeat clinical skills examination (post-test). Both pre- and post-testing was organized in 30-minute increments during which 5 learners were tested simultaneously with each learner being evaluated by one trainer at their own practice-and-testing station. Consistent with the mastery model, participants who did not meet or exceed the MPS would then complete additional deliberate practice and retesting until the MPS was achieved. After post-testing, participants completed a course evaluation questionnaire. A random sample of one-third of pre- and post-test clinical skills examinations were video-recorded and rescored to assess inter-rater agreement between instructors.

We conducted the NTHC SBML course using the CentraLineMan™ (Simulab, Seattle, WA, USA) simulator that provides a realistic representation of the anatomy of the right upper torso and head. This simulator has IJ and subclavian (SC) veins and carotid and SC arteries. In addition, the simulator features an arterial pulse, different coloured venous and arterial ‘blood’, appropriate venous and arterial blood pressures and realistic tissues with self-sealing veins and skin into which needles, dilators and guidewires may be inserted. It is compatible with ultrasound so as to produce highly realistic ultrasound images. A portable ultrasound machine (SonoSite Inc., Chicago, IL, USA) was made available at each practice and testing station. In addition, NTHC kits, sterile gowns, sterile gloves, sterile drapes and sterile CVC-insertion trays (Cardinal Health, LLC., McGaw Park, IL, USA) were available in unopened packaging at each practice and testing station.

To ensure standardization of training and grading of checklist items, faculty instructors (EC, CE, JJP, MS, RM) underwent a four-hour ‘train-the-trainers’ session led by an instructor with extensive NTHC SBML experience (JHB) the day before the trainees’ precourse. During this ‘train-the-trainers’ session, instructors reviewed the SBML curriculum and approaches to providing feedback, and practiced scoring simulated NTHC-insertions using the 28-item checklist.

### Measurement

A 28-item skills checklist was used to complete clinical skills evaluations (Table 1). This was adapted from a previously published NTHC-insertion skills checklist [9]. Each skill or action required for safe IJ NTHC-insertion was listed in order, given equal weight and scored dichotomously (done correctly/done incorrectly). The MPS was previously established at 79% by a multidisciplinary panel [9]. If the SC approach was used, the carotid artery was punctured or more than 2 needle passes (punctures of the skin) occurred, the simulation was terminated and the remaining checklist items were marked incorrect.

**Table 1:** **Temporary dialysis catheter insertion checklist: percentage of participants who performed each checklist item correctly**

**ITEM**	**Pretest (%) n = 22**	**Posttest (%) n = 17**
Informed consent obtained	77.3	88.2
Benefits (must state 1)		
Risks (must state 2)		
Consent given		
Washes hands	36.4	88.2
Places the patient in slight Trendelenburg position	22.7	88.2
Tests each port and flushes the lines with sterile saline	63.6	100
Clamps each port (may leave distal port open)	68.2	100
Area cleaned with chlorhexidine in a back and forth motion for at least 30 seconds	4.5	100
Gets in sterile gown, gloves, mask and cap	81.8	100
Area draped in usual fashion using full body drape	50.0	100
Ultrasound probe is properly set up, draped, and sonographic gel is used	59.1	88.2
The vein is localized using anatomical landmarks with the ultrasound machine	86.4	100
The skin is anesthetized with 1% lidocaine in a small wheal	81.8	100
The deeper structures are anesthetized	77.3	94.1
Using the large needle- (or catheter-) syringe complex, the vein is cannulated while aspirating (must be done with ultrasound)	59.1	100
Syringe is removed from the needle and the needle is held steady	63.6	94.1
The guidewire is advanced into the vein no more than 10-20 cm	22.7	100
The skin is knicked where the guidewire enters the skin (using a scalpel)	68.2	88.2
The dilator is advanced over the guidewire and the vein is dilated.	72.7	100
The catheter is advanced over the guidewire (with the guidewire held steady while catheter is moved forward)	59.1	100
Never lets go of the guidewire	54.5	100.0
As soon as line is in place, the guidewire is removed in its entirety	68.2	100.0
The line was advanced 14-16 cm (any other measurement is wrong)	31.8	100.0
Ensures there is blood flow from each port and flushes each port with sterile saline	50.0	88.2
Line is secured in place	54.5	100.0
Locks both ports with heparin or citrate	40.9	94.1
Places dressing over line or verbalize this	45.5	88.2
Requests a chest x-ray to confirm location	54.5	94.1
Notifies or places order that line is ok to use	45.5	100.0
Maintained sterile technique throughout	50.0	100.0

To assess inter-rater agreement, video-recorded clinical skills evaluations were rescored by one of two authors (JHB, JJP) who were blinded to pre- or post-testing status and the original learners checklist score.

### Statistical analysis

We evaluated differences between pre- and post-test scores using the Wilcoxon signed-rank test to assess the impact of the intervention. Spearman’s rank correlation coefficients were calculated to assess relationships between demographic factors, prior procedural experience and self-reported procedural-confidence with pre-test scores. The difference between pre-test scores of nephrology fellows versus those of the other trainees was assessed using the Wilcoxon rank-sum test. Inter-rater agreement was assessed by evaluating the percentage of items upon which the two raters agreed. A Kappa coefficient was not calculated because the checklist was previously validated and there was complete agreement on several items. We evaluated differences between the original scores of the video-recorded clinical evaluations with the scores obtained through video review using the Wilcoxon rank-sum test. We performed all statistical analyses using IBM SPSS version 22 (Chicago, IL).

## Results

**Figure 1:**
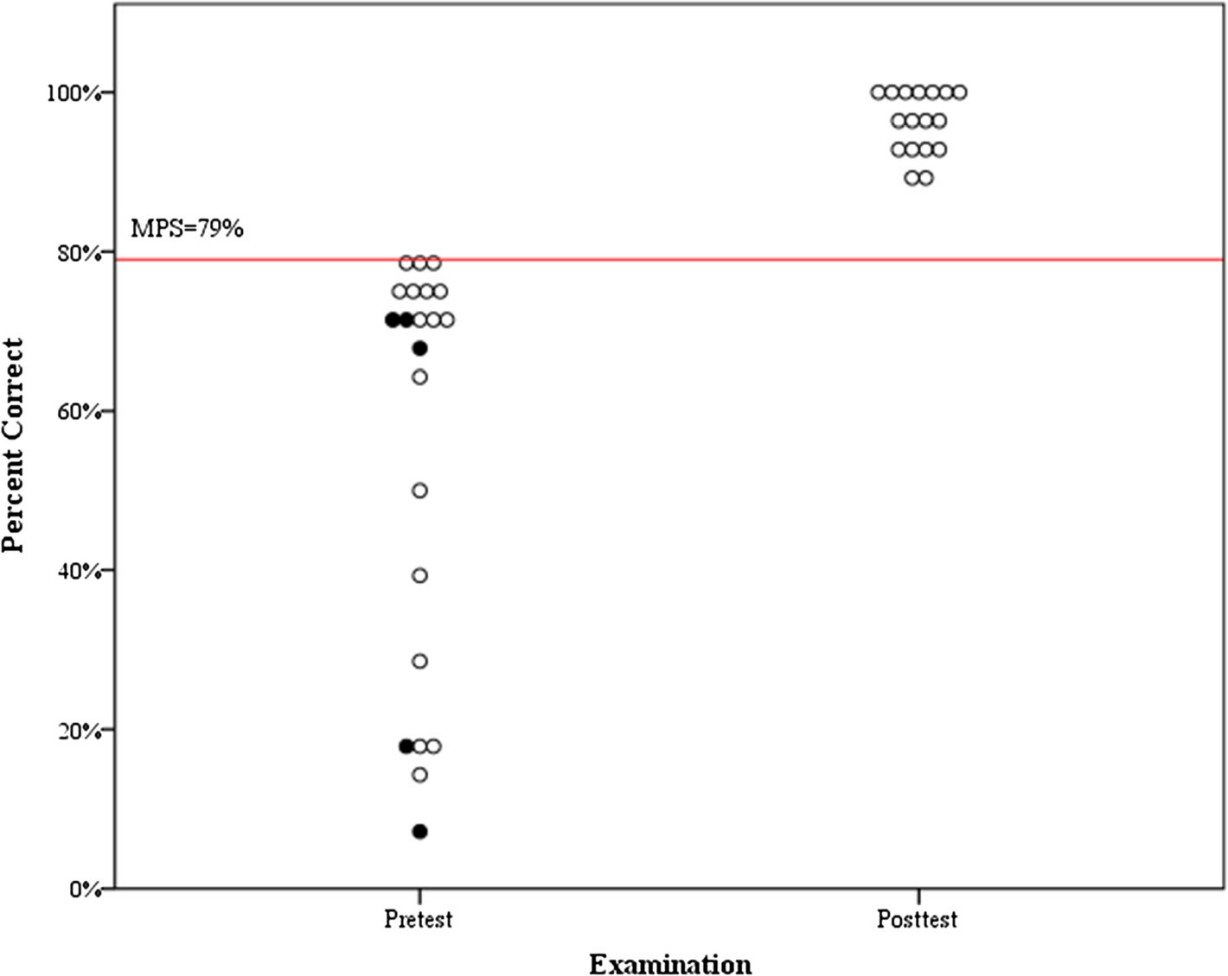
**Clinical skills examination (checklist) performance by 22 participants before simulation-based training and 17 participants after simulation-based training.** Minimum passing score (MPS) = 79%. Shaded circles represent the 5 participants who only underwent pretesting.

Twenty-two participants (11 nephrology fellows, 10 internal medicine residents and 1 medical student) enrolled in the precourse and completed pre-testing and training. Table 2 reports demographics, NTHC-insertion experience and procedural self-confidence. The median pre-test score was 20 (IQR, 7.25 to 21) checklist items correct out of a possible of 28 with no participants attaining the MPS. Seventeen participants participated in post-testing sessions on Day 2. The median post-test number of correct items increased by 7 (*p* = 0.001) to 27 (IQR, 26 to 28) with all participants achieving the MPS on their first attempt. Figure 1 depicts participants’ pre- and post-test clinical skills examination performance. The percentage of participants who performed each of the 28 checklist items correctly is detailed in Table 1.

**Table 2:** **Baseline demographics, prior experience and procedural self-confidence (n = 22)**

**Characteristic**		
Mean age (years)		31.1 ± 3.5
Female gender		10 (45.5)
Post-graduate year (PGY) of medical training	0	1 (4.5)
	1	2 (9.1)
	2	2 (9.1)
	3	6 (27.3)
	4	4 (18.2)
	5	6 (27.3)
	6	1 (4.5)
Nephrology fellows		11 (50)
Prior experience (number of previous IJ NTHC-insertions)	0-2	1
	3-5	3
	6-10	4
	11-15	2
	16-20	7
	≥ 21	5
Mean procedural self-confidence [0 to 100]		67.7 ± 21.5

Note: Values are expressed as mean ± SD or number (%).

The 5 participants who did not return for post-testing were nephrology fellows who each reported performing at least 20 prior IJ NTHC-insertions in clinical practice. Their median pre-test score of 19 (IQR, 3.5 to 20) was similar to overall group performance. Overall, no significant difference in pre-test scores were observed for nephrology fellows (*n* = 11) compared with trainees who had less medical training (*n* = 11): the fellows’ median score was 14 (IQR, 5 to 21) compared to 19 (IQR, 3.5 to 20) for the residents and student (*p* = 0.44).

Spearman’s rank correlation coefficients demonstrated no significant correlations between pre-test scores and age (*r =* -0.19*; p* = 0.40), gender (*r =* -0.42*; p* = 0.06), years of post-graduate medical training (*r =* -0.31*; p* = 0.89), clinical experience (*r* = 0.90*; p* = 0.69) or procedural self-confidence (*r* = 0.17*; p* = 0.45).

For the evaluations video-recorded in order to assess inter-rater agreement (*n* = 13), raters agreed on 331/364 (91%) of the rescored checklist items. The difference between the median of scores obtained by initial grading (22 (IQR, 20.5 to 27.5)) and the median of scores obtained from video review (23 (IQR, 18.5 to 27)) was not significant (*p* = 0.88).

Of the 17 participants who completed a post-training survey, 14 ‘strongly agreed’ (5/5 on the Likert scale) and 3 ‘agreed’ (4/5 on the Likert scale) that SBML-training improved their ability to competently insert NTHCs. As well, 15 ‘strongly agreed’ and 2 ‘agreed’ that this type of training should be a required component of nephrology fellowship training.

## Discussion

This study shows that SBML can be used to train current and future nephrology fellows in NTHC insertion at a national CME meeting. Consistent with the findings of earlier research [9], this program significantly improved trainee NTHC-insertion skills. No participant met or exceeded the MPS at baseline, however all met or exceeded the MPS on their first attempt after the educational intervention. Participants strongly endorsed that SBML improved their NTHC-insertion skills and that training of this type should be a required component of nephrology fellowship.

We believe that one of the strengths of this training intervention was the maintenance of a high level of realism during pre-testing, post-testing and deliberate-practice sessions. Anecdotally, many participants commented that they had received ‘partial’ simulation training for NTHC-insertion in the past, typically involving the use of a patient-simulator and ultrasound machine to repeatedly insert the finder needle into the IJ vein. Forcing participants to complete the entire procedure, using all necessary supplies in their original packaging, allowed them to encounter and overcome difficulties they will face in clinical practice but not during less realistic simulations: for example, the challenge of maintaining sterile technique while preparing the cover for the ultrasound probe only becomes evident during highly realistic simulation.

In addition to failing to achieve the MPS at pre-test, most trainees committed potentially serious errors. Notably, over-advancing the guidewire (beyond 20 cm) can provoke serious ventricular arrhythmias, particularly in patients with acute kidney injury [11]. This is a concerning finding as nearly all participants indicated having inserted at least 3 NTHCs in clinical practice before completing the simulation-based intervention. This confirms the findings of a recent survey in which many Canadian nephrology trainees reported inserting NTHCs despite feeling inadequately trained to do so [2]. Taken together, results of this study and others [2,8,9] suggest that providing SBML-training for NTHC insertion to current and incoming nephrology fellows at the annual CSN meeting could improve patient-safety. We suggest that training be mandatory because procedural self-confidence and prior experience inserting NTHCs did not correlate with proficiency.

SBML has many potential clinical benefits for Canadian nephrology training programs. SBML for CVC insertion reduces the number of needle passes per insertion [12,13], arterial punctures [13], line malpositions [13], insertion failures [13] and central line-associated bloodstream infections (CLABSI) [14]. Nonetheless, some experts have suggested that it may be prohibitively time-consuming and labour-intensive to require training, of any type, for NTHC-insertion, across all nephrology training programs [15]. Our findings suggest that a national CME meeting can be an effective venue to train a relatively large group of trainees from multiple centres, thus allowing training resources to be pooled. While there is evidence to suggest that substantial savings might result from reduced rates of CLABSIs [16,17], additional costs associated with conducting this type of training at a conference include accommodation expenses, flights, shipping costs, and costs related to trainers’ travel time. These additional costs may be mitigated somewhat if a large number of potential trainers and trainees plan to attend a conference for reasons other the training course alone. Companies may also be more willing to donate supplies for training in the context of a national meeting. As such, the longer-term feasibility of providing this type of training to nephrology fellows from across Canada annually would depend upon the results of a formal evaluation of its cost-effectiveness which was beyond the scope of this study. In the case of this study, all supplies and trainers’ time was donated leaving only travel expenses. This limits the generalizability of our findings with respect to providing similar programs at future conferences and in other settings. Training of all Canadian nephrology fellows at a CME meeting might not be necessary as some trainees may be able to receive similar training in a more cost-effective manner at their local training sites. SMBL-training for NTHC-insertion could be particularly helpful for filling in gaps in this training on a national level, particularly for nephrology fellows from centres where SMBL-training for NTHC-insertion may not be offered. One particular advantage of SBML training is that is enables standardization of procedure competence across many types of learners (e.g. nephrology fellows, nephrologists, internists) working in different settings.

This study has several other important limitations. The small sample size and self-selection of participants limit the generalizability of our findings. It is unknown if those who participated are more or less likely to be proficient at NTHC-insertion than their colleagues who did not elect to participate. We did not assess skill retention although this has been reported elsewhere [8]. Additional long-term studies are required to better define the duration for which procedural skills are retained following SBML, particularly when these skills are not used frequently in clinical practice. Use of a CME setting may allow for this important study at future CSN meetings. Finally, 5 of the 22 (23%) participants did not return for scheduled post-tests. The need for participants to devote a relatively large block of time to NTHC-insertion training while other conference events were taking place may be responsible for this finding. Because pretest scores and self-confidence were similar between those who dropped out and those who completed the program, we strongly believe SBML training should be mandatory.

## Conclusions

NTHC-insertion SBML during a national CME conference is an effective method to train current and future nephrology fellows. The long-term feasibility of providing this type of training at a national conference on a recurring basis would depend upon a formal evaluation of its cost effectiveness. Most participants reported having significant experience previously inserting NTHCs. However, pretest performance on a clinical skills examination was poor. SBML should be considered for incoming nephrology fellows prior to performing actual NTHC-insertions on patients.

## Abbreviations

SBML: Simulation-based-mastery-learning
NTHC: Non-tunneled temporary hemodialysis catheter
MPS: Minimum passing score
SD: Standard deviation
IQR: Interquartile range
CME: Continuing medical education
CSN: Canadian Society of Nephrology
IJ: Internal jugular
SC: Subclavian
CLABSI: Central line-associated bloodstream infection
